# Predictive value of left ventricular myocardial constructive work in patients with heart failure with preserved ejection fraction and preclinical diastolic dysfunction

**DOI:** 10.1186/s44348-025-00053-6

**Published:** 2025-08-08

**Authors:** Aram Chilingaryan, Lusine Tunyan, Milena Arzumanyan, Hovik Balyan

**Affiliations:** 1 Yerevan MSC, Yerevan, Armenia; 2 Yerevan State Medical University after Mkhitar Heratsi, Yerevan, Armenia

**Keywords:** Myocardial Work, Heart Failure with Preserved Ejection Fraction, Preclinical Diastolic Dysfunction, Diastolic dysfunction

## Abstract

**Background:**

We aimed to find predictors of ejection fraction (EF) deterioration in heart failure with preserved EF (HFpEF) patients to prevent their further deterioration.

**Methods:**

We studied 215 patients (mean age, 73 ± 8 years; 63% women) with HFpEF and with records of Charlson Comorbidity Index, glomerular filtration rate. Myocardial work, global longitudinal, radial, circumferential, and area strain. The global work index, global constructive work (GCW), wasted work, global work efficiency was obtained by echocardiography. Patients were followed up for 3 years.

**Results:**

Five patients developed myocardial infarction and were excluded from the study. Baseline EF was higher in female patients (61.2% ± 3.1% vs. 56.4% ± 2.7%, *P* < 0.002), in patients aged > 70 years (62.4% ± 2.1% vs. 57.1% ± 2.3%, *P* < 0.005), and in patients with end-diastolic volume index < 60 mL/m^2^ (56.1% ± 3.2% vs. 63.4% ± 2.3%, *P* < 0.001). EF decline compared to baseline was –7.3% ± 1.6% (*P* < 0.01). EF decline was significantly more in patients aged > 70 years, in patients with coronary artery disease and did not relate to sex, left ventricle size, cardiac index, and glomerular filtration rate. During follow-up 58 patients (27%) had EF < 50%, worsening in area strain (–27.9% ± 8.5% vs. –24.7% ± 5.3%, *P* < 0.003), global longitudinal strain (–19.7% ± 2.4% vs. –17.1% ± 1.6%, *P* < 0.005), and GCW (2,378% ± 117% vs. 2,102% ± 10%, *P* < 0.002). Patients with EF < 50% at the end of the study had less area strain and GCW baseline values compared with patients with EF > 50% (22.4% ± 7.2% vs. –27.6% ± 8.1%, *P* < 0.002; 2,081 ± 92 vs. 2,489 ± 127, *P* < 0.001). GCW was the predictor of EF deterioration (area under curve, 0.8853).

**Conclusions:**

GCW predicts EF decline in HFpEF patients which may help identify this subset of patients and prevent their further deterioration earlier.

## Background

Heart failure (HF) is a worldwide health problem with high morbidity and mortality. Its prevalence has been increased over time, mainly due to increase in share of HF with preserved ejection fraction (HFpEF) in overall HF syndrome, because of increased life expectancy and the emergence of age related comorbidities which in turn predispose to HFpEF [1].

HFpEF is remained an unresolved issue for years regarding its management. Proven therapies for HF with reduced ejection fraction (HFrEF) did not demonstrate benefit in this patient category.

Recently sodium-glucose cotransporter 2 (SGLT2) inhibitors have been proven to reduce hospitalization in patients with HFpEF [2]. Despite advances in the treatment of HFpEF, its management remains challenging. The benefits of SGLT2 inhibitors across the full range of ejection fraction (EF) [3] and of sacubitril/valsartan up to the lower end of preserved EF < 57% [4] suggest that, in some patients with HFpEF, certain pathophysiological mechanisms of HFrEF may coexist. Consequently, some subsets of HFpEF patients, particularly those with a gradual decline in EF over time, may benefit from established HFrEF therapies. Changes in EF are not uncommon in patients with heart failure, and many transition between HF categories over time [5].

Given the challenges in treating HFpEF, preventing its occurrence by targeting earlier stages of preclinical diastolic dysfunction (PDD), which often precedes HFpEF, might be effective. Additionally, little is known about the clinical course of PDD and its likelihood of progressing to HFpEF.

We aimed to assess the clinical course of patients with PDD and determine the time course of EF changes and the predictors of EF deterioration in both HFpEF and PDD patients, as well as predictors of PDD progression to HFpEF. This approach assumes that earlier and more aggressive treatment in these patient subsets might help prevent further deterioration.

## Methods

### Study population

All patients with dyspnea admitted to our hospital between 2019 and 2020 were evaluated for HFpEF. Diagnosis of HFpEF was made based on the HFA-PEFF algorithm [6]. Patients with arterial hypertension (AH) admitted at that time interval were assessed for PDD. The diagnosis of AH was based on the patient's history and the prescription of antihypertensive medications. PDD was identified by echocardiography. Exclusion criteria included poor acoustic window, age over 80 years, acute coronary syndrome at the time of admission, significant valvular heart disease (moderate to severe regurgitation or stenosis), atrial fibrillation, constrictive pericarditis, and hypertrophic cardiomyopathy. A total of 215 patients (mean age, 71 ± 8 years; 63% women) with HFpEF and 194 AH patients with PDD and excellent echo acoustic windows were enrolled.

Patients were followed up for 180 ± 24 days. The follow-up echocardiography was conducted at baseline and subsequently throughout the 3-year study period. All patients underwent follow-up echocardiography at regular intervals during this period. However, specific details on the median duration and interquartile range for the follow-up echocardiography were not provided. Additionally, no patients were excluded from follow-up, meaning that all patients received the scheduled assessments within the 3-year follow-up window. For more precise data on the timing and frequency of these assessments, further clarification would be needed from the study protocol or patient records.

### Baseline patient characteristics

Hyperlipidemia was defined based on the patient's serum lipid profile. Diabetes mellitus was identified by fasting blood glucose levels or the use of hypoglycemic medications. Chronic obstructive pulmonary disease was identified from previous medical records. AH was defined by office measurements if an average systolic blood pressure > 140 mmHg or diastolic blood pressure > 90 mmHg after three measurements. Prior myocardial infarction (MI) was identified from previous medical records. Coronary artery disease (CAD) was defined as a history of MI, percutaneous coronary intervention, or coronary artery bypass grafting. Anemia was defined as hemoglobin < 13 g/dL in men and < 12 g/dL in women. Estimated glomerular filtration rate (eGFR) was calculated using the specified estimation equation [7]. Body mass index was calculated from the patient's height and weight at the time of enrollment. The degree of comorbidity was assessed using the Charlson Comorbidity Index [8]. PDD was defined as elevated left-sided filling pressures with normal N-terminal pro–brain natriuretic peptide levels in asymptomatic patients. The E/A ratio and E/e'ratio were calculated, which helped determine that the left-sided filling pressures were elevated. Normal values for the E/A ratio are > 1.0, while normal E/e'is < 8. Elevated E/e'values (> 14) and an E/A ratio of < 0.8 suggest the presence of elevated left-sided filling pressures. Elevated filling pressures were defined if two of the following criteria were met: E/e’ average > 14, left atrial volume index > 34 mL/m^2^, left atrial strain < 20%, tricuspid regurgitation maximal velocity > 2.8 m/sec. Prescriptions of β-blockers, angiotensin-converting enzyme inhibitors (ACEI), angiotensin receptor blockers (ARB), and mineralocorticoid receptor antagonists prior to enrollment in the study were recorded.

### Echocardiography

Echocardiography was performed by two experienced echocardiographers using a GE Vivid 7 Ultrasound System (GE Healthcare). All linear and volumetric measurements, as well as the assessment of left ventricular filling pressures, were conducted according to joint recommendations from the American Society of Echocardiography and the European Association of Cardiovascular Imaging [9, 10]. Three-dimensional (3D) images were obtained through full-volume acquisition from four cardiac cycles with the patient holding their breath for optimal visualization. All images were stored and analyzed offline using EchoPac ver. 203 (GE Healthcare).

Speckle tracking was performed offline using Q-analysis, which involved manually tracing the endocardium in three apical and three parasternal short-axis views. The software automatically delineated the left ventricular walls, and manual corrections were made if necessary. The program then generated values for longitudinal strain (LS), circumferential strain (CS), radial strain, and left ventricular (LV) twist.

The global work index (GWI) was derived from pressure-strain loops obtained through 2D speckle tracking analysis, multiplied by brachial blood pressure measured immediately before the echocardiography exam with the patient in the left lateral decubitus position. Global constructive work (GCW) was calculated as the sum of positive work due to myocardial shortening during systole and negative work due to lengthening during isovolumic relaxation. Global wasted work represented energy loss due to myocardial lengthening in systole and shortening during isovolumic relaxation. Global work efficiency was defined as the percentage ratio of constructive work to the sum of constructive work and wasted work. EF and area strain (AS) were calculated in 3D using LVQ analysis on GE Vivid Ultrasound System and EchoPAC ver. 203.

### Statistical analysis

Statistical analysis was conducted using SAS ver. 9.2.1 (SAS Institute), with a significance level set at P < 0.05. Baseline clinical variables were summarized as means with standard deviations, medians with interquartile ranges for variables with skewed distributions, or as frequencies. Group differences were analyzed using t-tests for continuous variables and chi-square tests for categorical variables.

To evaluate the longitudinal changes in EF, linear mixed-effects regression models were employed, allowing for the fitting of a linear regression line for each individual. The cohort results were aggregated to assess changes in EF over time. Only age and LV end-diastolic diameter were analyzed as continuous variables within the model, while other variables were expressed categorically to simplify interpretation.

## Results

Patients with HFpEF have a higher prevalence of CAD, diabetes mellitus, anemia, and lower eGFR, as well as a lower prevalence of smokers compared with patients with PDD. HFpEF patients were predominantly women. The number of smokers was lower in the HFpEF group (Table 1).

**Table 1:** Baseline patient characteristics

Characteristic	HFpEF (*n* = 215)	PDD (*n* = 194)	*P*-value
Age (yr)	71 ± 8	64 ± 7	< 0.03
Female sex	144 (67.0)	101 (52.1)	< 0.05
Current smoker	39 (18.1)	60 (30.9)	< 0.01
Arterial hypertension	155 (72.1)	194 (100)	< 0.002
Diabetes mellitus	69 (32.1)	35 (18.0)	< 0.01
Hyperlipidemia	90 (41.9)	79 (40.7)	0.32
Coronary artery disease	82 (38.1)	63 (32.5)	0.24
Anemia	71 (33.0)	(13.5)	< 0.01
COPD	60 (27.9)	(17.6)	< 0.02
Cerebrovascular disease	88 (40.9)	(28.4)	< 0.01
EDVI (mL/m^2^)	60.0 ± 12.0	62.7 ± 11.0	0.38
eGFR (mL/min)	56.9 ± 21.7	62.3 ± 18.9	< 0.03
Charlson Comorbidity Index ≥ 3	81 (37.7)	35 (18.0)	< 0.01
ACEI/ARB	161 (74.9)	151 (77.8)	0.31
β-Blocker	137 (63.7)	120 (61.9)	0.21
MRA	146 (67.9)	118 (60.8)	0.18
NT-proBNP	586 (92)	186 (78)	0.001

Values are presented as mean ± standard deviation or number (%)

HFpEF, heart failure with preserved ejection fraction; PDD, preclinical diastolic dysfunction; COPD, chronic obstructive pulmonary disease; EDVI, end-diastolic volume index; eGFR, Estimated glomerular filtration rate; ACEI, angiotensin-converting enzyme inhibitors; ARB, angiotensin receptor blockers; MRA, mineralocorticoid receptor antagonists; NT-proBNP, N-terminal pro–brain natriuretic peptide

Mean baseline values of EF did not differ between the HFpEF and PDD groups, with values of 58.7% and 59.1%, respectively (Table 2). Baseline EF was higher in female patients, in patients over 70 years of age, in those with anemia, and in patients with an end-diastolic volume index < 60 mL/m^2^ in both groups. Five patients with HFpEF and two with PDD experienced MI during follow-up and were excluded from the study. Additionally, echocardiographic follow-up data were unavailable for 7 patients with HFpEF and 10 patients with PDD, and these patients were also excluded. During follow-up, 39 patients (20%) with PDD developed HFpEF.

**Table 2:** EF values at baseline relative to patients’ characteristics

Baseline parameter	HFpEF		PDD	
	EF (%)	*P*-value	EF (%)	*P*-value
Mean EF (%)	58.7 ± 3.1		59.1 ± 3.2	0.08
Sex		0.002		0.003
Female (*n* = 144)	61.2 ± 3.1		63.4 ± 3.8	
Male (*n* = 71)	56.4 ± 2.7		57.2 ± 2.7	
Age (yr)		0.005		0.03
> 70 (*n* = 112)	62.4 ± 2.1		64.1 ± 3.4	
< 70 (*n* = 103)	56.7 ± 2.3		55.2 ± 2.8	
Coronary artery disease		0.12		0.18
Yes (*n* = 82)	58.9 ± 2.9		58.3 ± 3.1	
No (*n* = 133)	57.6 ± 3.3		59.2 ± 3.2	
Median EDVI (mL/m^2^)		0.001		0.001
< 60 (*n* = 87)	63.4 ± 2.3		64.1 ± 3.1	
≥ 60 (*n* = 128)	56.1 ± 3.2		56.9 ± 2.9	
Charlson Comorbidity Index		0.23		0.08
≥ 3 (*n* = 90)	59.2 ± 3.8		58.9 ± 3.4	
≤ 3 (*n* = 125)	58.1 ± 3.5		61.1 ± 3.7	
Median eGFR (mL/min)		0.28		0.23
< 56.9 (*n* = 76)	58.2 ± 3.1		59.8 ± 3.9	
< 56.9 (*n* = 139)	57.8 ± 3.5		58.8 ± 3.7	
Anemia		0.002		0.002
Yes (*n* = 71)	62.1 ± 3.8		64.3 ± 4.2	
No (*n* = 144)	56.9 ± 3.1		57.1 ± 3.5	

Values are presented as mean ± standard deviation

EF, ejection fraction; HFpEF, heart failure with preserved ejection fraction; PDD, preclinical diastolic dysfunction; EDVI, end-diastolic volume index; eGFR, Estimated glomerular filtration rate

The overall reduction in EF among HFpEF and PDD patients was 7.3% and 4.2%, respectively, over 3 years, and was statistically significant both within and between groups. The reduction in EF was more pronounced in patients over 70 years and in those with CAD, and was not associated with sex, LV size, Charlson Comorbidity Index, or eGFR in either group (Table 3).

**Table 3:** Changes in EF for 3 years follow-up

Baseline parameter	HFpEF		PDD	
	EF change (%)	P-value	EF change (%)	P-value
Overall EF change (%)	–7.3 ± 1.6	0.002	–4.2 ± 1.1^*^	0.04
Sex		0.34		0.45
Female (*n* = 142)	–7.0 ± 1.9		–4.5 ± 1.1^*^	
Male (*n* = 68)	–7.4 ± 2.1		–4.1 ± 1.3^*^	
Age (yr)		0.01		0.03
> 70 (*n* = 109)	–7.9 ± 1.8		–5.7 ± 1.8^*^	
< 70 (*n* = 101)	–5.7 ± 1.7		–3.9 ± 1.2^*^	
Coronary artery disease		0.001		0.03
Yes (*n* = 80)	–7.8 ± 1.9		–5.5 ± 1.9^*^	
No (*n* = 130)	–5.3 ± 1.6		–3.9 ± 1.6^*^	
Median EDVI (mL/m^2^)		0.53		0.44
< 60 (*n* = 85)	–6.8 ± 1.5		–4.4 ± 1.3^*^	
≥ 60 (*n* = 125)	–7.1 ± 1.8		–4.1 ± 1.2^*^	
Charlson Comorbidity Index		0.76		0.53
≥ 3 (*n* = 87)	–7.2 ± 1.9		–4.2 ± 1.5^*^	
≤ 3 (*n* = 123)	–7.1 ± 1.7		–4.4 ± 1.3^*^	
Median eGFR (mL/min)		0.47		0.41
< 56.9 (*n* = 73)	–6.9 ± 1.4		–4.6 ± 1.7^*^	
< 56.9 (*n* = 137)	–7.2 ± 2.1		–4.2 ± 1.4^*^	
Anemia		0.72		0.62
Yes (*n* = 71)	–7.3 ± 1.4		–4.4 ± 1.6^*^	
No (*n* = 144)	–7.2 ± 1.5		–4.1 ± 1.4^*^	

Values are presented as mean ± standard deviation

EF, ejection fraction; HFpEF, heart failure with preserved ejection fraction; PDD, preclinical diastolic dysfunction; EDVI, end-diastolic volume index; eGFR, Estimated glomerular filtration rate

^*^P < 0.05 (statistically significant between groups)

We observed significant reduction in AS, LS, and GCW both within and between groups (Table 4). There was a correlation of EF with LS, AS, GWI, and GCW in both groups (Table 5).

**Table 4:** Changes in global strain values and myocardial work

Parameter	HFpEF			PDD		
	Baseline	3 years FU	P-value	Baseline	3 years FU	*P*-value
Longitudinal strain (%)	–19.7 ± 2.4	–17.1 ± 1.6	0.005	–20.2 ± 3.1^*^	–18.3 ± 2.1^*^	0.02
Circumferential strain (%)	–14.3 ± 3.5	–13.9 ± 3.1	0.18	–16.1 ± 2.9	–15.7 ± 2.1	0.23
Radial strain (%)	31.7 ± 10.8	29.8 ± 10.1	0.06	34.8 ± 9.3	32.1 ± 8.9	0.12
Left ventricular twist (%)	1.9 ± 0.8	1.8 ± 0.9	0.150	2.1 ± 0.7	1.9 ± 0.8	0.21
Area strain (%)	–27.9 ± 8.5	–24.7 ± 5.3	0.003	29.4 ± 8.7^*^	27.3 ± 9.2^*^	0.01
Global work index (mg%)	2,470 ± 161	2,210 ± 147	0.005	2,581 ± 187	2,436 ± 168	0.02
Global constructive work (mg%)	2,378 ± 117	2,102 ± 101	0.002	2,493 ± 137^*^	2,342 ± 124^*^	0.02
Global wasted work (mg%)	112 ± 9	116 ± 7	0.07	95 ± 8	103 ± 9	0.08
Global work efficiency (%)	95 ± 2	94 ± 3	0.21	98 ± 5	97 ± 6	0.30

Values are presented as mean ± standard deviation

HFpEF, heart failure with preserved ejection fraction; PDD, preclinical diastolic dysfunction; FU, follow-up

^*^P < 0.05 (statistically significant between groups)

**Table 5:** Correlation of deformation and myocardial work parameter with EF

Variable	HFpEF				PDD			
	LS	AS	GWI	GCW	LS	AS	GWI	GCW
EF	0.43	0.52	0.51	0.59	0.41	0.49	0.48	0.52
P-value	0.01	< 0.001	0.003	< 0.001	0.02	0.003	0.003	< 0.001

EF, ejection fraction; HFpEF, heart failure with preserved ejection fraction; PDD, preclinical diastolic dysfunction; LS, longitudinal strain; AS, area strain; GWI, global work index; GCW, global constructive work

During follow-up, 58 patients (27%) with HFpEF and 27 patients (14%) with PDD had an EF < 50%. Patients with an EF < 50% in both groups at the end of the study had significantly lower baseline values of AS and GCW compared with patients with an EF > 50% (Table 6).

**Table 6:** Baseline parameters of patients who consequently had EF < 50% or > 50% at the end of the study

Parameter	HFpEF			PDD		
	EF < 50%	EF > 50%	*P*-value	EF < 50%	EF > 50%	*P*-value
Longitudinal strain (%)	–18.1 ± 2.2	–20.2 ± 2.4	0.01	–19.1 ± 2.4^*^	–21.3 ± 3.1^*^	0.01
Circumferential strain (%)	–13.4 ± 3.8	–14.5 ± 4.1	0.04	–15.2 ± 3.3^*^	–17.1 ± 3.9^*^	0.04
Radial strain (%)	31.4 ± 11.2	31.8 ± 10.6	0.12	31.9 ± 12.1	32.6 ± 11.4	0.18
Left ventricular twist (%)	1.9 ± 0.9	1.9 ± 1.2	0.17	2.1 ± 1.0	2.3 ± 1.3	0.19
Area strain (%)	23.9 ± 7.2	–28.8 ± 8.1	0.002	24.6 ± 8.3^*^	–29.6 ± 9.1^*^	0.008
Global work index (mg%)	2,451 ± 142	2,493 ± 158	0.06	2,489 ± 158	2,546 ± 165	0.05
Global constructive work (mg%)	2,081 ± 92	2,489 ± 127	< 0.001	2,249 ± 101^*^	2,591 ± 118^*^	0.002
Global wasted work (mg%)	118 ± 9	111 ± 8	0.06	106 ± 10	113 ± 11	0.08
Global work efficiency (%)	94.6 ± 2.1	95.7 ± 1.8	0.05	92.3 ± 2.9	92.1 ± 2.4	0.12

Values are presented as mean ± standard deviation

EF, ejection fraction; HFpEF, heart failure with preserved ejection fraction; PDD, preclinical diastolic dysfunction

^*^P < 0.05 (statistically significant between groups)

**Fig. 1:**
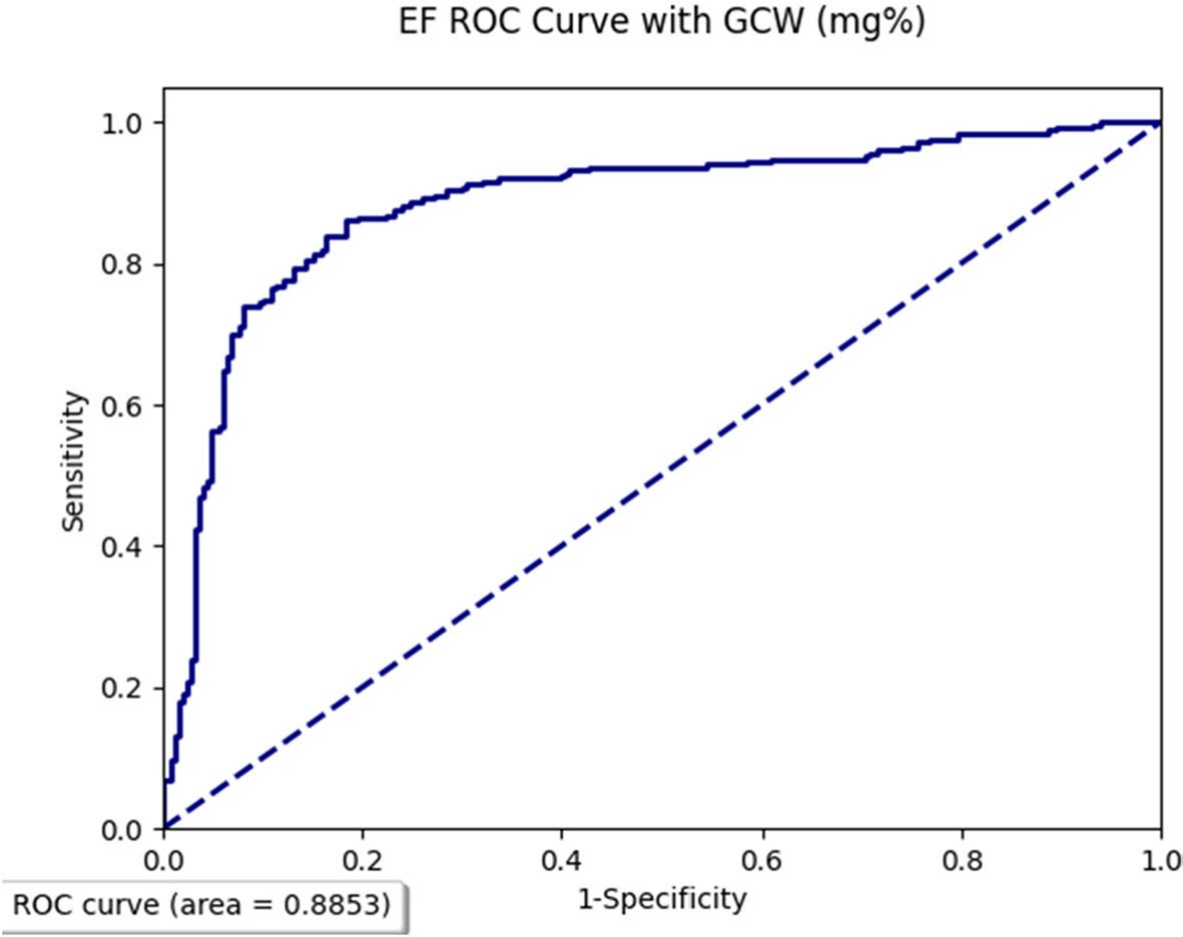
Ejection fraction receiver operating characteristic (ROC) curve with global constructive work (GCW)

GCW was the only predictor of EF deterioration in patients with HFpEF (area under curve, 0.8853) (Fig. 1, Table 7).

**Table 7:** Predictive values of deformation and myocardial work parameters for ejection fraction deterioration in patients with heart failure with preserved ejection fraction

Parameters	AUC	95% CI	*P*-value
Longitudinal strain (%)	0.6348	0.5404–0.7292	0.0597
Circumferential strain (%)	0.5839	0.4873–0.6805	0.0883
Radial strain (%)	0.6184	0.5232–0.7136	0.0621
Left ventricular twist (%)	0.5841	0.4875–0.6807	0.0847
Area strain (%)	0.6972	0.6071–0.7873	0.0503
Global work index (mg%)	0.6483	0.5547–0.7419	0.0602
Global constructive work (mg%)	0.8853	0.8228–0.9478	< 0.001
Global wasted work (mg%)	0.6235	0.5285–0.7185	0.0654
Global work efficiency (%)	0.6872	0.5963–0.7781	0.0510

AUC, area under the curve; CI, confidence interval

We could not identify predictors of EF reduction in patients with PDD in the multivariate analysis. However, patients with PDD who developed HFpEF had lower absolute values of LS, CS, AS, and GCW (Table 8). AS and GCW were predictors of PDD transformation to HFpEF (Table 9).

**Table 8:** Baseline parameters of patients with PDD who consequently developed HFpEF or remained in PDD state

Parameter	PDD	HFpEF	*P*-value
Longitudinal strain (%)	–18.1 ± 2.2	–20.2 ± 2.4	0.01
Circumferential strain (%)	–13.4 ± 3.8	–14.5 ± 4.1	0.04
Radial strain (%)	31.4 ± 11.2	31.8 ± 10.6	0.12
Left ventricular twist (%)	1.9 ± 0.9	1.9 ± 1.2	0.17
Area strain (%)	–23.9 ± 7.2	–28.8 ± 8.1	0.002
Global work index (mg%)	2,451 ± 142	2,493 ± 158	0.06
Global constructive work (mg%)	2,081 ± 92	2,489 ± 127	< 0.001
Global wasted work (mg%)	118 ± 9	111 ± 8	0.06
Global work efficiency (%)	94.6 ± 2.1	95.7 ± 1.8	0.05

Values are presented as mean ± standard deviation

HFpEF, heart failure with preserved ejection fraction; PDD, preclinical diastolic dysfunction

**Table 9:** Predictive values of deformation and myocardial work parameters for PDD transformation to HFpEF in patients with PDD

Parameter	AUC	95% CI	*P*-value
Longitudinal strain (%)	0.5861	0.5161–0.6561	0.2495
Circumferential strain (%)	0.5342	0.4642–0.6042	0.2071
Radial strain (%)	0.5139	0.4439–0.5839	0.3515
Left ventricular twist (%)	0.5443	0.4743–0.6143	0.3452
Area strain (%)	0.7988	0.7588–0.8388	0.0266
Global work index (mg%)	0.5328	0.4628–0.6028	0.2932
Global constructive work (mg%)	0.8572	0.8172–0.8972	0.0149
Global wasted work (mg%)	0.5413	0.4713–0.6113	0.4431
Global work efficiency (%)	0.5194	0.4494–0.5894	0.2549

AUC, area under the curve; CI, confidence interval; HFpEF, heart failure with preserved ejection fraction; PDD, preclinical diastolic dysfunction

## Discussion

We conducted this study to assess dynamics of systolic function in patients with HFpEF and PDD as well as the clinical course of patients with PDD. We observed significant reduction in systolic function parameters in patients with HPrEF and PDD, with average EF reduction of 7.3% and 4.2%, respectively, over 3 years follow-up. About one-third of our patients with HFpEF had EF < 50% during follow-up of which 12% had EF < 40% at the end of the study. Patients with PDD have less reduction of EF compared to HFpEF patients, but we observed reduction of EF < 50% in 14% of these patients.

Our results shows that PDD is not a benign condition and requires close attention. We speculate that these patients might require the same treatment as patients with HFpEF to prevent transformation of PDD to HFpEF.

We observed that patients with PDD exhibit dynamics in several parameters that resemble those seen in patients with HFpEF, albeit to a lesser extent. Furthermore, during follow-up, 20% of patients initially diagnosed with PDD progressed to HFpEF. Therefore, PDD may be considered an early stage of HFpEF, suggesting that preventive measures should commence early in the continuum of heart failure.

Although HFpEF and its precursor, PDD, are predominantly characterized by diastolic dysfunction, there are numerous pathophysiological factors that contribute to concomitant systolic dysfunction. [11].

Although several mechanisms of systolic dysfunction have been proposed, including the development of MI, neurohormonal activation, and infiltrative and inflammatory processes, the exact mechanism remains unclear. We excluded patients with new MI from the study, so MI cannot explain the EF decline observed in our study. Additionally, Dunlay et al. [12] showed that neurohormonal modulator medications did not alter the course of EF decline in patients with HFpEF.

In our study, changes in EF were not affected by baseline LV dimensions, baseline EF, or Charlson Comorbidity Index, suggesting that mechanisms other than initial systolic functional status, neurohormonal activation, and inflammation may be involved in the development of systolic dysfunction.

We observed a decline in LS, CS, and AS in patients with EF reduction. These parameters positively correlated with EF; however, GCW was the only predictor of EF decline in patients with HFpEF. Additionally, both AS and GCW were predictors of the transformation of PDD to HFpEF.

GCW appears to be a sensitive parameter for predicting subsequent EF decline, likely because it reflects LV endocardial constructive shortening in relation to afterload. Given that endocardial function deteriorates earlier than radial function—which is more closely associated with EF—a reduced GCW may serve as an early indicator of impending radial dysfunction and global systolic decline.

The results of the study enable us to predict which patients might develop HFpEF and experience a later decline in EF, allowing for more aggressive early treatment. Although the study by Dunlay et al. [12] did not demonstrate a benefit from ACEI/ARB and β-blockers, it remains unclear how these patients would respond to newer HF medications, such as sacubitril/valsartan. They might also gain more benefit from SGLT2 inhibitors while still in the normal EF range, compared with their counterparts at a lower risk of EF deterioration.

## Conclusions

We speculate that patients with HFpEF and PDD who have an increased risk of systolic function deterioration might benefit more from HFrEF evidence-based treatments, which could be initiated before EF declines. Although data from the PARAGON-HF trial showed that angiotensin receptor-neprilysin inhibitors (ARNI) are effective in patients with HFpEF with EF up to 57% [4], our study found that EF decline was not related to baseline EF values. Even patients with higher EF and lower GCW were at risk of EF decline and might potentially benefit from ARNI. Further studies are needed to explore this question.

## Data Availability

No datasets were generated or analysed during the current study.

## Abbreviations

2D: Two-dimensional
3D: Three-dimensional
ACEI: Angiotensin-converting enzyme inhibitors
AH: Arterial hypertension
ARB: Angiotensin receptor blockers
ARNI: Angiotensin receptor-neprilysin inhibitors
AS: Area strain
CAD: Coronary artery disease
CS: Circumferential strain
EF: Ejection fraction
eGFR: Estimated glomerular filtration rate
GCW: Global constructive work
GWI: Global work index
HF: Heart failure
HFpEF: Heart failure with preserved ejection fraction
HFrEF: Heart failure with reduced ejection fraction
LS: Longitudinal strain
LV: Left ventricular
MI: Myocardial infarction
PDD: Preclinical diastolic dysfunction
SGLT2: Sodium-glucose cotransporter 2
